# 1,5-Bis[1-(4-bromo­phen­yl)ethyl­idene]thio­carbonohydrazide

**DOI:** 10.1107/S1600536813009720

**Published:** 2013-04-13

**Authors:** Zhiqing Gao

**Affiliations:** a Dongchang College, Liaocheng University, Liaocheng 250059, People’s Republic of China

## Abstract

The asymmetric unit of the title compound, C_17_H_16_Br_2_N_4_S, contains two independent mol­ecules in which the benzene rings form dihedral angles of 20.0 (1) and 55.3 (1)°. In the crystal, a pair of N—H⋯S hydrogen bonds link the two different independent mol­ecules into a dimer.

## Related literature

For the crystal structures of related compounds, see: Feng *et al.* (2011 ▶); Zhao (2011 ▶); Schmitt *et al.* (2011 ▶).
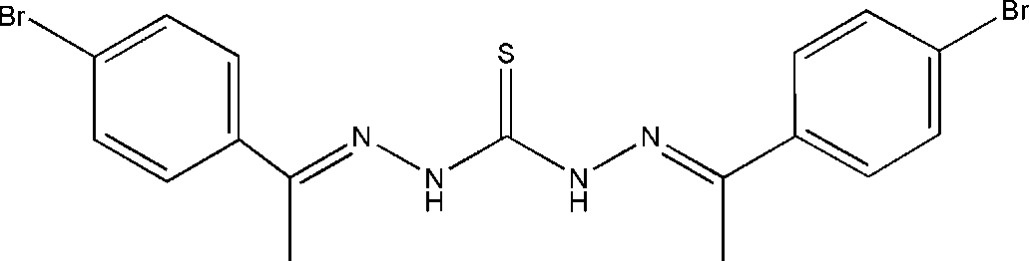


## Experimental

### 

#### Crystal data

C_17_H_16_Br_2_N_4_S
*M* *_r_* = 468.22
Triclinic, 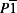

*a* = 11.5800 (11) Å
*b* = 13.6950 (12) Å
*c* = 14.2860 (13) Å
α = 118.401 (2)°
β = 90.977 (1)°
γ = 108.404 (1)°
*V* = 1852.6 (3) Å^3^
*Z* = 4
Mo *K*α radiation
μ = 4.49 mm^−1^
*T* = 298 K
0.43 × 0.37 × 0.33 mm

#### Data collection

Bruker SMART APEX CCD area-detector diffractometer
Absorption correction: multi-scan (*SADABS*; Sheldrick, 1996 ▶) *T*_min_ = 0.248, *T*_max_ = 0.319
9457 measured reflections
6456 independent reflections
2949 reflections with *I* > 2σ(*I*)
*R*_int_ = 0.081

#### Refinement

*R*[*F* ^2^ > 2σ(*F* ^2^)] = 0.073
*wR*(*F* ^2^) = 0.214
*S* = 0.96
6456 reflections
438 parameters
H-atom parameters constrained
Δρ_max_ = 0.90 e Å^−3^
Δρ_min_ = −1.33 e Å^−3^

### 

Data collection: *SMART* (Bruker, 2007 ▶); cell refinement: *SAINT* (Bruker, 2007 ▶); data reduction: *SAINT*; program(s) used to solve structure: *SHELXS97* (Sheldrick, 2008 ▶); program(s) used to refine structure: *SHELXL97* (Sheldrick, 2008 ▶); molecular graphics: *SHELXTL* (Sheldrick, 2008 ▶); software used to prepare material for publication: *SHELXTL*.

## Supplementary Material

Crystal structure: contains datablock(s) I, global. DOI: 10.1107/S1600536813009720/cv5398sup1.cif

Structure factors: contains datablock(s) I. DOI: 10.1107/S1600536813009720/cv5398Isup2.hkl

Supplementary material file. DOI: 10.1107/S1600536813009720/cv5398Isup3.cml

Additional supplementary materials:  crystallographic information; 3D view; checkCIF report

## Figures and Tables

**Table 1:** Hydrogen-bond geometry (Å, °)

*D*—H⋯*A*	*D*—H	H⋯*A*	*D*⋯*A*	*D*—H⋯*A*
N5—H5*B*⋯S1	0.86	2.71	3.551 (8)	168
N1—H1⋯S2	0.86	2.69	3.551 (8)	174

**Fig. 1.**
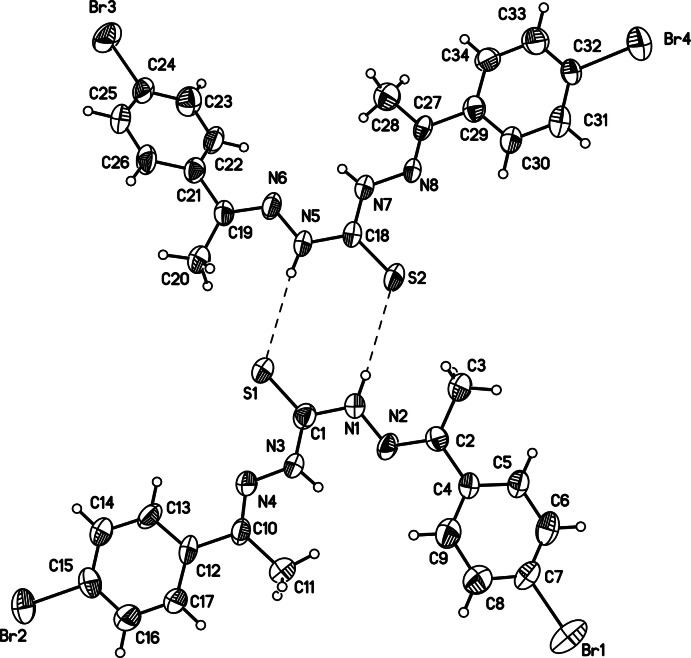
A hydrogen-bonded (dashed lines) dimer in the asymmetric unit of (I) showing the atomic numbering and 50% probability displacement ellipsoids

## supplementary crystallographic information

### Comment

In a continuation of our structural study of thiocarbonohydrazides (Feng *et al.*, 2011), we present here the title compound (I).

In (I) (Fig. 1), all bond lengths and angles are normal and correspond to those observed in the related thiocarbonohydrazides (Feng *et al.*, 2011; Zhao, 2011; Schmitt *et al.*, 2011). The benzene rings C4—C9 and C12—C17 form a dihedral angle of 20.0 (1)°, while benzene rings C21—C26 and C29—C34 form a dihedral angle of 55.3 (1)°.

In the crystal, intermolecular N—H···S hydrogen bonds link two independent molecules into dimer (Fig. 1).

### Experimental

*p*-Br-Acetophenone (1.0 mmol) and thiocarbohydrazide (0.5 mmol) were mixed in 25 ml flash in the methanol. After 6 h stirring at 354 K, the resulting mixture was cooled to room temperature, and recrystalized from ethanol, and afforded the title compound as a crystalline solid.

### Refinement

All H atoms were placed in geometrically idealized positions (N—H 0.86 and C—H 0.93–0.96 Å) and treated as riding on their parent atoms, with *U*_iso_(H) = 1.2–1.5 *U*_eq_(C) (C, N).

### Crystal data

**Table:**

C_17_H_16_Br_2_N_4_S	*Z* = 4
*M**_r_* = 468.22	*F*(000) = 928
Triclinic, *P*1	*D*_x_ = 1.679 Mg m^−^^3^
*a* = 11.5800 (11) Å	Mo *K*α radiation, λ = 0.71073 Å
*b* = 13.6950 (12) Å	Cell parameters from 2095 reflections
*c* = 14.2860 (13) Å	θ = 2.8–21.2°
α = 118.401 (2)°	µ = 4.49 mm^−^^1^
β = 90.977 (1)°	*T* = 298 K
γ = 108.404 (1)°	Yellow, block
*V* = 1852.6 (3) Å^3^	0.43 × 0.37 × 0.33 mm

### Data collection

**Table:**

Bruker SMART APEX CCD area-detector diffractometer	6456 independent reflections
Radiation source: fine-focus sealed tube	2949 reflections with *I* > 2σ(*I*)
Graphite monochromator	*R*_int_ = 0.081
phi and ω scans	θ_max_ = 25.0°, θ_min_ = 2.3°
Absorption correction: multi-scan (*SADABS*; Sheldrick, 1996)	*h* = −7→13
*T*_min_ = 0.248, *T*_max_ = 0.319	*k* = −16→14
9457 measured reflections	*l* = −16→16

### Refinement

**Table:**

Refinement on *F*^2^	Secondary atom site location: difference Fourier map
Least-squares matrix: full	Hydrogen site location: inferred from neighbouring sites
*R*[*F*^2^ > 2σ(*F*^2^)] = 0.073	H-atom parameters constrained
*wR*(*F*^2^) = 0.214	*w* = 1/[σ^2^(*F*_o_^2^) + (0.1033*P*)^2^] where *P* = (*F*_o_^2^ + 2*F*_c_^2^)/3
*S* = 0.96	(Δ/σ)_max_ = 0.001
6456 reflections	Δρ_max_ = 0.90 e Å^−^^3^
438 parameters	Δρ_min_ = −1.33 e Å^−^^3^
0 restraints	Extinction correction: *SHELXL97* (Sheldrick, 2008), Fc^*^=kFc[1+0.001xFc^2^λ^3^/sin(2θ)]^-1/4^
Primary atom site location: structure-invariant direct methods	Extinction coefficient: 0.0038 (8)

### Special details

**Table:**

Geometry. All e.s.d.'s (except the e.s.d. in the dihedral angle between two l.s. planes) are estimated using the full covariance matrix. The cell e.s.d.'s are taken into account individually in the estimation of e.s.d.'s in distances, angles and torsion angles; correlations between e.s.d.'s in cell parameters are only used when they are defined by crystal symmetry. An approximate (isotropic) treatment of cell e.s.d.'s is used for estimating e.s.d.'s involving l.s. planes.
Refinement. Refinement of *F*^2^ against ALL reflections. The weighted *R*-factor *wR* and goodness of fit *S* are based on *F*^2^, conventional *R*-factors *R* are based on *F*, with *F* set to zero for negative *F*^2^. The threshold expression of *F*^2^ > σ(*F*^2^) is used only for calculating *R*-factors(gt) *etc*. and is not relevant to the choice of reflections for refinement. *R*-factors based on *F*^2^ are statistically about twice as large as those based on *F*, and *R*- factors based on ALL data will be even larger.

### Fractional atomic coordinates and isotropic or equivalent isotropic displacement parameters (Å^2^)

**Table:**

	*x*	*y*	*z*	*U*_iso_*/*U*_eq_	
Br1	0.86326 (12)	1.60584 (12)	1.59389 (9)	0.0846 (5)	
Br2	1.15040 (11)	0.55332 (10)	0.95650 (10)	0.0669 (4)	
Br3	0.32652 (11)	0.37660 (11)	−0.03711 (8)	0.0723 (4)	
Br4	−0.09677 (12)	1.33393 (11)	0.64953 (10)	0.0787 (5)	
N5	0.4538 (7)	0.8654 (6)	0.6079 (6)	0.045 (2)	
H5B	0.4943	0.8559	0.6519	0.055*	
N6	0.4306 (7)	0.7886 (6)	0.4978 (6)	0.046 (2)	
N7	0.3534 (7)	0.9606 (6)	0.5655 (6)	0.049 (2)	
H7	0.3550	0.9157	0.4987	0.058*	
N8	0.2919 (7)	1.0377 (6)	0.5921 (6)	0.047 (2)	
S2	0.4295 (3)	1.0494 (3)	0.7779 (2)	0.0688 (9)	
C18	0.4121 (9)	0.9556 (8)	0.6457 (7)	0.045 (2)	
C19	0.4609 (8)	0.6957 (8)	0.4612 (7)	0.041 (2)	
C20	0.5183 (9)	0.6633 (9)	0.5305 (7)	0.056 (3)	
H20A	0.6005	0.7214	0.5670	0.084*	
H20B	0.5226	0.5860	0.4861	0.084*	
H20C	0.4688	0.6615	0.5834	0.084*	
C21	0.4303 (8)	0.6187 (8)	0.3412 (7)	0.042 (2)	
C22	0.4345 (9)	0.6678 (8)	0.2735 (8)	0.052 (3)	
H22	0.4588	0.7501	0.3053	0.062*	
C23	0.4042 (9)	0.5988 (9)	0.1630 (8)	0.057 (3)	
H23	0.4086	0.6328	0.1196	0.069*	
C24	0.3668 (8)	0.4769 (9)	0.1180 (7)	0.047 (3)	
C25	0.3645 (9)	0.4233 (9)	0.1799 (8)	0.051 (3)	
H25	0.3407	0.3409	0.1470	0.061*	
C26	0.3982 (9)	0.4952 (8)	0.2910 (8)	0.050 (3)	
H26	0.3998	0.4610	0.3335	0.060*	
C27	0.2443 (9)	1.0463 (7)	0.5156 (8)	0.044 (2)	
C28	0.2551 (10)	0.9804 (9)	0.3973 (8)	0.061 (3)	
H28A	0.3316	0.9665	0.3933	0.091*	
H28B	0.2540	1.0277	0.3651	0.091*	
H28C	0.1865	0.9053	0.3588	0.091*	
C29	0.1686 (8)	1.1214 (8)	0.5468 (7)	0.041 (2)	
C30	0.1683 (9)	1.1928 (8)	0.6561 (8)	0.051 (3)	
H30	0.2209	1.1971	0.7091	0.061*	
C31	0.0914 (10)	1.2570 (9)	0.6868 (9)	0.058 (3)	
H31	0.0928	1.3050	0.7600	0.069*	
C32	0.0137 (9)	1.2497 (8)	0.6096 (8)	0.048 (3)	
C33	0.0121 (10)	1.1817 (9)	0.5017 (9)	0.061 (3)	
H33	−0.0413	1.1780	0.4497	0.073*	
C34	0.0898 (10)	1.1187 (9)	0.4703 (8)	0.053 (3)	
H34	0.0896	1.0736	0.3968	0.064*	
C1	0.6961 (9)	0.9711 (8)	0.9249 (7)	0.044 (2)	
C2	0.6406 (8)	1.2301 (8)	1.1112 (7)	0.042 (2)	
C3	0.5534 (10)	1.2427 (9)	1.0443 (8)	0.061 (3)	
H3A	0.5938	1.2585	0.9922	0.091*	
H3B	0.5283	1.3077	1.0904	0.091*	
H3C	0.4816	1.1702	1.0071	0.091*	
C4	0.6932 (8)	1.3211 (8)	1.2269 (7)	0.043 (2)	
C5	0.6906 (8)	1.4327 (8)	1.2704 (7)	0.044 (2)	
H5A	0.6540	1.4526	1.2269	0.053*	
C6	0.7420 (9)	1.5178 (9)	1.3795 (8)	0.056 (3)	
H6	0.7399	1.5939	1.4082	0.068*	
C7	0.7958 (9)	1.4894 (9)	1.4448 (7)	0.051 (3)	
C8	0.7975 (10)	1.3767 (10)	1.4055 (8)	0.066 (3)	
H8	0.8305	1.3562	1.4504	0.079*	
C9	0.7471 (10)	1.2936 (10)	1.2942 (8)	0.059 (3)	
H9	0.7500	1.2177	1.2649	0.071*	
C10	0.9075 (9)	0.9240 (8)	1.0604 (7)	0.042 (2)	
C11	0.9352 (11)	1.0257 (10)	1.1749 (8)	0.076 (4)	
H11A	0.9116	1.0871	1.1756	0.114*	
H11B	1.0225	1.0573	1.2043	0.114*	
H11C	0.8893	0.9976	1.2182	0.114*	
C12	0.9696 (8)	0.8369 (8)	1.0347 (7)	0.042 (2)	
C13	0.9539 (9)	0.7456 (8)	0.9292 (7)	0.050 (3)	
H13	0.9045	0.7403	0.8736	0.060*	
C14	1.0085 (10)	0.6628 (9)	0.9037 (8)	0.056 (3)	
H14	0.9979	0.6037	0.8320	0.067*	
C15	1.0793 (9)	0.6687 (9)	0.9861 (9)	0.050 (3)	
C16	1.0998 (10)	0.7599 (10)	1.0913 (8)	0.062 (3)	
H16	1.1509	0.7664	1.1465	0.074*	
C17	1.0443 (10)	0.8407 (10)	1.1137 (8)	0.061 (3)	
H17	1.0573	0.9010	1.1854	0.073*	
S1	0.6625 (3)	0.8667 (2)	0.79389 (19)	0.0573 (8)	
N1	0.6438 (7)	1.0560 (6)	0.9636 (6)	0.046 (2)	
H1	0.5879	1.0546	0.9220	0.055*	
N2	0.6838 (7)	1.1437 (7)	1.0710 (6)	0.045 (2)	
N3	0.7716 (7)	0.9799 (7)	1.0024 (6)	0.051 (2)	
H3	0.7833	1.0366	1.0681	0.061*	
N4	0.8306 (7)	0.9031 (7)	0.9815 (6)	0.046 (2)	

### Atomic displacement parameters (Å^2^)

**Table:**

	*U*^11^	*U*^22^	*U*^33^	*U*^12^	*U*^13^	*U*^23^
Br1	0.0993 (10)	0.0729 (10)	0.0402 (7)	0.0114 (7)	0.0123 (6)	0.0105 (6)
Br2	0.0741 (8)	0.0582 (8)	0.0797 (9)	0.0427 (6)	0.0312 (6)	0.0316 (6)
Br3	0.0858 (9)	0.0716 (9)	0.0357 (6)	0.0361 (7)	0.0156 (5)	0.0051 (6)
Br4	0.0866 (9)	0.0617 (9)	0.0857 (9)	0.0481 (7)	0.0081 (7)	0.0234 (7)
N5	0.060 (5)	0.032 (5)	0.037 (5)	0.026 (4)	0.007 (4)	0.007 (4)
N6	0.059 (5)	0.031 (5)	0.038 (5)	0.022 (4)	0.011 (4)	0.008 (4)
N7	0.064 (6)	0.038 (5)	0.036 (5)	0.030 (4)	0.004 (4)	0.006 (4)
N8	0.067 (6)	0.035 (5)	0.034 (5)	0.032 (4)	0.003 (4)	0.006 (4)
S2	0.112 (3)	0.0566 (19)	0.0339 (15)	0.0562 (17)	0.0061 (14)	0.0046 (13)
C18	0.056 (6)	0.031 (6)	0.042 (6)	0.023 (5)	0.006 (5)	0.009 (5)
C19	0.049 (6)	0.035 (6)	0.041 (6)	0.023 (5)	0.012 (4)	0.015 (5)
C20	0.074 (7)	0.052 (7)	0.039 (6)	0.037 (6)	0.013 (5)	0.011 (5)
C21	0.040 (6)	0.038 (6)	0.043 (6)	0.018 (5)	0.012 (4)	0.014 (5)
C22	0.070 (7)	0.031 (6)	0.043 (6)	0.020 (5)	0.007 (5)	0.009 (5)
C23	0.074 (8)	0.056 (8)	0.048 (7)	0.029 (6)	0.023 (5)	0.028 (6)
C24	0.037 (6)	0.044 (7)	0.043 (6)	0.023 (5)	0.013 (4)	0.006 (5)
C25	0.062 (7)	0.034 (6)	0.050 (7)	0.024 (5)	0.015 (5)	0.014 (5)
C26	0.057 (7)	0.025 (6)	0.062 (7)	0.014 (5)	0.010 (5)	0.019 (5)
C27	0.053 (6)	0.017 (5)	0.045 (6)	0.010 (4)	−0.002 (5)	0.006 (4)
C28	0.067 (7)	0.065 (8)	0.051 (7)	0.033 (6)	0.013 (5)	0.025 (6)
C29	0.044 (6)	0.030 (6)	0.049 (6)	0.011 (4)	0.011 (4)	0.021 (5)
C30	0.067 (7)	0.043 (6)	0.041 (6)	0.030 (5)	0.009 (5)	0.013 (5)
C31	0.071 (8)	0.039 (7)	0.056 (7)	0.029 (6)	0.015 (6)	0.014 (5)
C32	0.059 (7)	0.030 (6)	0.053 (7)	0.024 (5)	0.000 (5)	0.016 (5)
C33	0.072 (8)	0.042 (7)	0.066 (8)	0.021 (6)	−0.004 (6)	0.026 (6)
C34	0.071 (7)	0.037 (6)	0.045 (6)	0.018 (5)	−0.008 (5)	0.017 (5)
C1	0.051 (6)	0.032 (6)	0.041 (6)	0.013 (5)	0.007 (5)	0.015 (5)
C2	0.041 (6)	0.046 (6)	0.042 (6)	0.022 (5)	0.010 (4)	0.020 (5)
C3	0.076 (8)	0.068 (8)	0.039 (6)	0.046 (6)	0.017 (5)	0.016 (5)
C4	0.050 (6)	0.032 (6)	0.042 (6)	0.020 (5)	0.008 (4)	0.013 (5)
C5	0.056 (7)	0.033 (6)	0.041 (6)	0.020 (5)	0.015 (4)	0.015 (5)
C6	0.066 (7)	0.031 (6)	0.060 (7)	0.015 (5)	0.020 (6)	0.015 (5)
C7	0.063 (7)	0.040 (7)	0.034 (6)	0.014 (5)	0.012 (5)	0.009 (5)
C8	0.085 (9)	0.068 (9)	0.048 (7)	0.036 (7)	0.015 (6)	0.026 (6)
C9	0.075 (8)	0.057 (7)	0.049 (7)	0.034 (6)	0.019 (5)	0.021 (6)
C10	0.061 (7)	0.031 (6)	0.036 (6)	0.024 (5)	0.016 (5)	0.013 (5)
C11	0.105 (9)	0.067 (8)	0.043 (6)	0.050 (7)	−0.008 (6)	0.007 (6)
C12	0.044 (6)	0.027 (5)	0.045 (6)	0.016 (4)	0.015 (4)	0.009 (5)
C13	0.065 (7)	0.041 (6)	0.027 (5)	0.019 (5)	0.008 (4)	0.006 (5)
C14	0.073 (8)	0.049 (7)	0.041 (6)	0.034 (6)	0.016 (5)	0.011 (5)
C15	0.046 (6)	0.047 (7)	0.066 (7)	0.026 (5)	0.019 (5)	0.030 (6)
C16	0.073 (8)	0.064 (8)	0.043 (7)	0.037 (6)	0.007 (5)	0.016 (6)
C17	0.085 (8)	0.063 (8)	0.035 (6)	0.049 (6)	0.009 (5)	0.011 (5)
S1	0.081 (2)	0.0479 (17)	0.0348 (14)	0.0368 (15)	0.0041 (12)	0.0074 (12)
N1	0.055 (5)	0.038 (5)	0.041 (5)	0.026 (4)	0.005 (4)	0.013 (4)
N2	0.050 (5)	0.047 (5)	0.032 (4)	0.028 (4)	0.017 (4)	0.009 (4)
N3	0.074 (6)	0.037 (5)	0.035 (5)	0.030 (4)	0.002 (4)	0.006 (4)
N4	0.052 (5)	0.046 (5)	0.038 (5)	0.030 (4)	0.009 (4)	0.012 (4)

### Geometric parameters (Å, º)

**Table:**

Br1—C7	1.902 (9)	C33—H33	0.9300
Br2—C15	1.885 (10)	C34—H34	0.9300
Br3—C24	1.918 (9)	C1—N3	1.333 (11)
Br4—C32	1.903 (10)	C1—N1	1.367 (11)
N5—C18	1.342 (11)	C1—S1	1.667 (9)
N5—N6	1.374 (10)	C2—N2	1.310 (11)
N5—H5B	0.8600	C2—C4	1.478 (12)
N6—C19	1.292 (11)	C2—C3	1.482 (12)
N7—C18	1.363 (11)	C3—H3A	0.9600
N7—N8	1.366 (10)	C3—H3B	0.9600
N7—H7	0.8600	C3—H3C	0.9600
N8—C27	1.285 (11)	C4—C5	1.360 (12)
S2—C18	1.666 (9)	C4—C9	1.388 (13)
C19—C21	1.484 (12)	C5—C6	1.394 (13)
C19—C20	1.484 (12)	C5—H5A	0.9300
C20—H20A	0.9600	C6—C7	1.373 (14)
C20—H20B	0.9600	C6—H6	0.9300
C20—H20C	0.9600	C7—C8	1.376 (14)
C21—C26	1.397 (12)	C8—C9	1.412 (13)
C21—C22	1.410 (13)	C8—H8	0.9300
C22—C23	1.364 (13)	C9—H9	0.9300
C22—H22	0.9300	C10—N4	1.282 (11)
C23—C24	1.378 (13)	C10—C12	1.486 (12)
C23—H23	0.9300	C10—C11	1.501 (13)
C24—C25	1.388 (13)	C11—H11A	0.9600
C25—C26	1.375 (13)	C11—H11B	0.9600
C25—H25	0.9300	C11—H11C	0.9600
C26—H26	0.9300	C12—C17	1.382 (13)
C27—C29	1.474 (13)	C12—C13	1.386 (12)
C27—C28	1.522 (13)	C13—C14	1.374 (13)
C28—H28A	0.9600	C13—H13	0.9300
C28—H28B	0.9600	C14—C15	1.378 (13)
C28—H28C	0.9600	C14—H14	0.9300
C29—C34	1.391 (12)	C15—C16	1.374 (13)
C29—C30	1.395 (13)	C16—C17	1.363 (14)
C30—C31	1.378 (13)	C16—H16	0.9300
C30—H30	0.9300	C17—H17	0.9300
C31—C32	1.357 (13)	N1—N2	1.372 (10)
C31—H31	0.9300	N1—H1	0.8600
C32—C33	1.366 (14)	N3—N4	1.350 (10)
C33—C34	1.374 (14)	N3—H3	0.8600
			
C18—N5—N6	118.9 (8)	N3—C1—N1	113.2 (8)
C18—N5—H5B	120.5	N3—C1—S1	125.1 (8)
N6—N5—H5B	120.5	N1—C1—S1	121.7 (7)
C19—N6—N5	119.3 (8)	N2—C2—C4	115.2 (8)
C18—N7—N8	119.5 (8)	N2—C2—C3	123.0 (8)
C18—N7—H7	120.3	C4—C2—C3	121.5 (8)
N8—N7—H7	120.3	C2—C3—H3A	109.5
C27—N8—N7	118.1 (8)	C2—C3—H3B	109.5
N5—C18—N7	113.3 (8)	H3A—C3—H3B	109.5
N5—C18—S2	122.5 (7)	C2—C3—H3C	109.5
N7—C18—S2	124.2 (7)	H3A—C3—H3C	109.5
N6—C19—C21	114.5 (8)	H3B—C3—H3C	109.5
N6—C19—C20	124.5 (8)	C5—C4—C9	118.4 (9)
C21—C19—C20	121.0 (8)	C5—C4—C2	121.7 (9)
C19—C20—H20A	109.5	C9—C4—C2	120.0 (9)
C19—C20—H20B	109.5	C4—C5—C6	120.8 (9)
H20A—C20—H20B	109.5	C4—C5—H5A	119.6
C19—C20—H20C	109.5	C6—C5—H5A	119.6
H20A—C20—H20C	109.5	C7—C6—C5	120.1 (9)
H20B—C20—H20C	109.5	C7—C6—H6	119.9
C26—C21—C22	117.5 (9)	C5—C6—H6	119.9
C26—C21—C19	121.3 (9)	C6—C7—C8	121.3 (9)
C22—C21—C19	121.1 (8)	C6—C7—Br1	119.5 (8)
C23—C22—C21	122.3 (9)	C8—C7—Br1	119.1 (8)
C23—C22—H22	118.8	C7—C8—C9	117.0 (10)
C21—C22—H22	118.8	C7—C8—H8	121.5
C22—C23—C24	117.7 (10)	C9—C8—H8	121.5
C22—C23—H23	121.2	C4—C9—C8	122.3 (10)
C24—C23—H23	121.2	C4—C9—H9	118.9
C23—C24—C25	122.8 (9)	C8—C9—H9	118.9
C23—C24—Br3	119.1 (8)	N4—C10—C12	116.0 (8)
C25—C24—Br3	117.9 (7)	N4—C10—C11	125.2 (9)
C26—C25—C24	118.3 (9)	C12—C10—C11	118.7 (8)
C26—C25—H25	120.9	C10—C11—H11A	109.5
C24—C25—H25	120.9	C10—C11—H11B	109.5
C25—C26—C21	121.3 (9)	H11A—C11—H11B	109.5
C25—C26—H26	119.4	C10—C11—H11C	109.5
C21—C26—H26	119.4	H11A—C11—H11C	109.5
N8—C27—C29	115.9 (9)	H11B—C11—H11C	109.5
N8—C27—C28	125.0 (9)	C17—C12—C13	116.2 (9)
C29—C27—C28	119.0 (8)	C17—C12—C10	122.4 (8)
C27—C28—H28A	109.5	C13—C12—C10	121.4 (9)
C27—C28—H28B	109.5	C14—C13—C12	122.5 (9)
H28A—C28—H28B	109.5	C14—C13—H13	118.8
C27—C28—H28C	109.5	C12—C13—H13	118.8
H28A—C28—H28C	109.5	C13—C14—C15	119.0 (10)
H28B—C28—H28C	109.5	C13—C14—H14	120.5
C34—C29—C30	117.6 (9)	C15—C14—H14	120.5
C34—C29—C27	122.3 (9)	C16—C15—C14	120.2 (9)
C30—C29—C27	119.9 (8)	C16—C15—Br2	119.1 (8)
C31—C30—C29	121.0 (9)	C14—C15—Br2	120.8 (8)
C31—C30—H30	119.5	C17—C16—C15	119.3 (10)
C29—C30—H30	119.5	C17—C16—H16	120.4
C32—C31—C30	119.6 (10)	C15—C16—H16	120.4
C32—C31—H31	120.2	C16—C17—C12	122.9 (10)
C30—C31—H31	120.2	C16—C17—H17	118.6
C31—C32—C33	121.1 (10)	C12—C17—H17	118.6
C31—C32—Br4	120.5 (8)	C1—N1—N2	117.6 (7)
C33—C32—Br4	118.4 (7)	C1—N1—H1	121.2
C32—C33—C34	119.7 (9)	N2—N1—H1	121.2
C32—C33—H33	120.1	C2—N2—N1	120.2 (8)
C34—C33—H33	120.1	C1—N3—N4	122.5 (8)
C33—C34—C29	120.9 (10)	C1—N3—H3	118.8
C33—C34—H34	119.5	N4—N3—H3	118.8
C29—C34—H34	119.5	C10—N4—N3	117.5 (8)

### Hydrogen-bond geometry (Å, º)

**Table:**

*D*—H···*A*	*D*—H	H···*A*	*D*···*A*	*D*—H···*A*
N5—H5*B*···S1	0.86	2.71	3.551 (8)	168
N1—H1···S2	0.86	2.69	3.551 (8)	174
